# Twelve-Month Outcomes Using Aflibercept 8 mg in Treatment-Naïve and Pretreated Diabetic Macular Edema: A Swiss Retina Research Network Report

**DOI:** 10.1016/j.xops.2026.101087

**Published:** 2026-01-22

**Authors:** Jan Spindler, Dmitri Artemiev, Isabel B. Pfister, Christin Schild, Anne Tillmann, Katharina Heck, Felix Gabathuler, Ludovico Ruscitti, Tahm Spitznagel, Konstantinos Kitsos-Kalyvianakis, Marion R. Munk, Katja Hatz, Sandrine A. Zweifel, Gabriela Grimaldi, Gábor Márk Somfai, Aude Ambresin, Andreas Weinberger, Chiara M. Eandi, Nicolas Feltgen, Alice Kitay, Andreas Ebneter, Justus G. Garweg

**Affiliations:** 1 Berner Augenklinik and Swiss Eye Institute, Bern, Switzerland; 2 Department of Ophthalmology, Kantonsspital St. Gallen, Switzerland; 3 Augenärzte Praxisgemeinschaft Gutblick AG, Pfaeffikon, Switzerland; 4 Vista Augenklinik Binningen, Binningen, Switzerland; 5 Department of Ophthalmology, University Hospital Zuerich, University of Zuerich, Switzerland; 6 Ente Ospedaliero Cantonale (EOC), Ophthalmology Clinic, and Institute of Clinical Neurosciences of Southern Switzerland (INSI) Lugano, Switzerland; 7 Department of Ophthalmology, Stadtspital Zuerich, Zuerich, Switzerland; 8 Spross Research Institute, Zuerich, Switzerland; 9 Swiss Visio Retina Research Center, Swiss Visio Montchoisi, Lausanne, Switzerland; 10 Department of Ophthalmology, Inselspital, University Hospital Bern, Bern, Switzerland; 11 Department of Ophthalmology, Feinberg School of Medicine, Northwestern University, Chicago, Illinois; 12 Faculty of Medicine, University of Basel, Basel, Switzerland; 13 Department of Ophthalmology, Semmelweis University, Budapest, Hungary; 14 Pallas-Klinik Olten, Olten, Switzerland; 15 Department of Ophthalmology, RWTH Aachen University, Aachen, Germany; 16 Jules-Gonin Eye Hospital, Fondation Asile des Aveugles, Department of Ophthalmology, University of Lausanne, Switzerland; 17 Department of Surgical Sciences, University of Torino, Italy; 18 tazz- Talacker Augen Zentrum Zürich, Zürich, Switzerland

**Keywords:** Diabetic macular edema, Anti-VEGF, Aflibercept 8mg, Treatment switch, Swiss Retina Research Network

## Abstract

**Purpose:**

To evaluate the effectiveness and safety of intravitreal aflibercept 8 mg (Afl 8) for the therapy of treatment-naïve and pretreated diabetic macular edema (DME) in a clinical routine setting.

**Design:**

A multicenter, retrospective cohort study of consecutive DME patients treated with Afl 8 over 12 months.

**Subjects:**

One hundred fifty-six eyes (124 patients) with DME, including 42 (26.9%) treatment-naïve and 114 (73.1%) pretreated eyes receiving Afl 8 for 1 year.

**Methods:**

Data from electronic medical records were collected retrospectively at 5 predefined time points. The primary outcomes were the mean changes in corrected visual acuity (VA), center-point retinal thickness (CRT), central subfield thickness (CST), treatment intervals, and adverse events (AEs). Secondary outcomes included the number of injections, persistent fluid, and treatment adherence. These parameters were recorded from the beginning of anti-VEGF treatment until switching occurred in pretreated eyes.

**Main Outcome Measures:**

Mean change in VA, CRT, CST, treatment intervals, and AEs.

**Results:**

In treatment-naïve eyes, VA improved from 72.9 ± 10.7 ETDRS letters at baseline to 77.7 ± 9.7 (*P* = 0.006); and from 73.9 ± 11.2 to 75.4 ± 10.1 ETDRS letters (*P* = 0.094) in pretreated eyes. Central subfield thickness decreased in both groups (naïve: 448.9 ± 154.3 μm to 320.0 ± 80.1 μm, *P* < 0.001; pretreated: 336.6 ± 90.5 μm to 310.2 ± 69.9 μm, *P* = 0.047). After 12 months, 38.1% of naïve eyes and 27.2% of pretreated eyes were free of retinal fluid in the central 1 mm. In treatment-naïve eyes, the mean treatment interval was 15.3 ± 12.0 weeks at 12 months. In pretreated eyes, the interval increased from 7.6 ± 3.7 weeks at the time of switching to 13.0 ± 9.0 weeks (*P* < 0.001). Two eyes (4.8%) in the naïve group and 16 eyes (14%) in the switcher group were switched away within the first year due to insufficient response to Afl 8 therapy. No AEs were reported in the treatment-naïve group. In the pretreated group, 3 cases of noninfectious intraocular inflammation (IOI; 1.9%; 1 recurrent), 2 instances of acute intraocular pressure rise, and 1 vitreous hemorrhage were reported.

**Conclusions:**

Afl 8 offers a promising approach to reducing the treatment burden in DME. It enables extended dosing intervals without compromising efficacy and safety, especially in refractory eyes. However, a possibly increased rate of mild IOI has been observed.

**Financial Disclosure(s):**

Proprietary or commercial disclosure may be found in the Footnotes and Disclosures at the end of this article.

Treating eyes with insufficiently responsive neovascular age-related macular degeneration (nAMD) and diabetic macular edema (DME), which have a high treatment demand, can be challenging in real life. Faricimab (Vabysmo, Roche), a bispecific anti-VEGF and anti-Ang-2 agent, has demonstrated a stronger drying potential than previously marketed drugs. Its use in clinical practice has confirmed the expectations from randomized clinical trials (RCTs) of this agent. Nevertheless, a significant number of eyes have not responded adequately. After more than a decade of favorable clinical experience with 2 mg of aflibercept, a high-dose of intravitreal aflibercept (8 mg in 70 μL, Eylea HD [Afl 8]) was developed with the intent to reduce the treatment burden while maintaining efficacy. The Candela Phase II RCT^1^ was the first to demonstrate the therapeutic benefits of Afl 8 compared with the standard aflibercept 2 mg dose in patients with nAMD, namely a reduced treatment burden without compromising safety. The PHOTON phase II/III RCT demonstrated that after a loading phase of 3 Afl 8 injections, the drug can be administered every 12 or 16 weeks. It is noninferior to the 2 mg dose administered every 8 weeks (after 5 monthly loading injections) over 48 weeks in terms of visual gains, without compromising the drug's safety profile.^2^

As of September 2025, only a few real-world case series have reported outcomes for Afl 8 in nAMD and DME for up to 6 months. Two observational studies reported effective disease control and prolonged treatment intervals in switched nAMD patients, though a considerable proportion of patients were unable to extend treatment intervals beyond 8 weeks.^3^^,^^4^ Four recent papers reported an increased incidence of intraocular inflammation (IOI) following Afl 8 injection, though without vision loss.^5^, ^6^, ^7^, ^8^ There have been no reports detailing clinical experience with this drug over 12 months in DME following its market access in the United States in August 2023 and in Europe and Switzerland in August 2024 since the Photon phase II/III RCT.^2^

If there are no relevant safety findings, Afl 8's improved drying potential and extended duration of effect would be particularly beneficial for diabetic patients with high treatment demands, as it would reduce their treatment burden. A possibly increased incidence of IOI after Afl 8 in nAMD and DME^5^^,^^6^ underscores the call for further real-life data.^9^ In this report, we present the first 1-year Afl 8 treatment outcomes in DME in a real-world setting across 12 Swiss centers under a treat-and-extend regimen for 12 months. We evaluated the responses of patients in terms of function and morphology, as well as treatment patterns and safety signals. This evaluation included both treatment-naïve patients and those who had been switched from other anti-VEGF treatments.

## Methods

This retrospective, longitudinal, multicenter study analyzed patient data from 12 major ophthalmology centers in Switzerland that are part of the Swiss Retina Research Network. Data were collected from August 2024 to September 2025 and included consecutive eyes treated with intravitreal aflibercept (8 mg/70 μL; Afl 8) injections with 1-year follow-up after commencing Afl 8 treatment. The study was approved by all involved ethics committees under the lead of the Ethics Committee of the Canton of Bern (project ID 2024-01026) based on general or study-specific consent from all included patients to use their coded data for this analysis. The study was conducted in accordance with the principles of the latest version of the Declaration of Helsinki. For patients who had signed earlier versions of general consent that did not fully conform to the current Swiss Human Research Act, attempts were made to obtain additional consent wherever possible (SR 810.30 — Federal Act on Research Involving Human Beings [Human Research Act, HRA] of September 30, 2011 [Status as of September 1, 2023]) (Accessed August 12, 2025; https://www.fedlex.admin.ch/eli/cc/2013/617/en).

Eligible participants were adults (≥18 years old) with treatment-naïve or pretreated DME who received intravitreal anti-VEGF therapy with Afl 8 for 12 (±1) months. The choice of drug and the decision to use Afl 8 treatment was at the discretion of the treating physicians. The following inclusion criteria were applied: Clinically significant DME requiring intravitreal therapy at the initiation of treatment; Snellen best-corrected visual acuity (VA) of at least 0.1 at diagnosis; and treatment-naïve or previous treatment with ranibizumab, aflibercept, bevacizumab, faricimab, or brolucizumab (switching between agents is allowed). Patients were excluded if any of the following applied: refusal to consent to the use of their coded data, dexamethasone injections within 6 months before the first Afl 8 injection, follow-up of less than 11 months, preexisting structural macular damage from any cause without functional potential (e.g., advanced age-related macular degeneration with loss of the ellipsoid zone or hypertransmission on OCT imaging), any systemic comorbidities interfering with treatment outcomes (e.g., local or systemic rheumatoid diseases or vasculitis requiring treatment), any opacifications in the optic axis relevant to visual function that could prohibit ocular imaging and fundoscopy, or any intraocular surgery or laser treatment within 3 months prior to inclusion, except for YAG laser capsulotomy. A history of vitrectomy more than 3 months prior to inclusion in this study was not explicitly excluded.

Generally, evidence-based treatment decisions were made by individual physicians. The goal was to eliminate retinal fluid, which is often unachievable, particularly for patients with chronic DME. The key criterion guiding treatment intensity and changes in treatment intervals was the change in retinal fluid. Central subfield thickness (CST) was assessed as part of the overall clinical evaluation, particularly in cases with an incomplete response. A 320 μm threshold for CST was used as a rule for fluid tolerance in cases that did not achieve a completely dry retina.

For this analysis, eyes that had been stable without therapy prior to developing new activity were categorized as treatment-naïve if they had not received an injection within 6 months before the baseline. The initial treatment interval for all pretreated eyes remained the same after the first Afl 8 injection. Data from electronic medical records were collected retrospectively at predefined time points: before initiating Afl 8 treatment (baseline) and 1, 3, 4, 6, and 12 months after initiating Afl 8 therapy. For pretreated eyes, the following time points were also evaluated: diagnosis and start of the first anti-VEGF treatment; 3 to 4 months after the initiation of the first anti-VEGF treatment; 6 months before switching; and the last injection before switching. Upon inclusion in this study, the best-corrected VA of each patient was recorded and compared to their prescription. The following were recorded at each time point: Snellen or spectacle-refracted VA, intraocular pressure, adverse events (AEs), and OCT imaging data, including center-point retinal thickness (CRT), CST, and the presence of intraretinal fluid (IRF), subretinal fluid (SRF), or both, in the foveal 1 mm circle of the ETDRS grid. All sites used the Heidelberg Spectralis device (Heidelberg Engineering) for OCT measurements using the follow-up function. Sites were also instructed on how to perform CRT and CST measurements according to a standard operating procedure after manually checking and correcting the segmentation, if necessary. For statistical purposes, Snellen VA values were converted to ETDRS letter scores,^10^ where 1.0 Snellen VA corresponded to 85 ETDRS letters.

The primary outcome measures were changes in VA (ETDRS letters), changes in CST and CRT (μm), and, in pretreated eyes, changes in treatment intervals (weeks) comparing the last treatment interval before the switch to the last treatment interval at the 12-month follow-up. Secondary outcomes included the number of eyes with an absence of retinal fluid in the central 1-mm ETDRS subfield of the macula after a single Afl 8 injection (fast dryers) and after 3 to 4, 6, and 12 months of Afl 8 treatment, respectively. Other secondary outcomes included the proportion of eyes with persistent IRF and/or SRF at these time points, the number of injections administered during the observation period, the proportion of eyes remaining on Afl 8 or switching to another drug, and the incidence of clinically relevant AEs. Data from patients who switched to another drug were censored after switching away from Afl 8.

All patients were treated according to a treat-and-extend protocol for fluid tolerance, accepting residual fluid if there was no further functional improvement after 2 consecutive injections and the CST was below 320 μm. For treatment-naïve patients, the loading phase included a second injection 1 month after the initial injection. Thereafter, treatment intervals were modified by 2-week decrements or increments in response to the evolution of CRT and CST, as determined by the treating physician's experience. Due to the retrospective nature of this study, strict criteria for treatment interval modification were not applied. For pretreated patients, the last treatment interval prior to switching was followed, and treatment modification thereafter followed the aforementioned fluid-tolerant treat-and-extend protocol.

Descriptive statistics, subgroup comparisons, and correlation analyses were performed. The Shapiro–Wilk test was used to test the distribution of data and revealed that the data were not normally distributed. Therefore, the data are presented as mean ± standard deviation as well as median and interquartile ranges (IQRs). Nonparametric tests were used for comparisons. Longitudinal changes in functional and morphological parameters from baseline to 12 months were analyzed using the Wilcoxon rank test. The Friedman test for dependent samples was used to compare multiple time points in the longitudinal analysis. The Mann–Whitney U test was applied to compare the functional and morphological outcomes of the naïve and switcher subgroups. The level of significance was set at *P* < 0.05. All statistical analyses were performed using SPSS Statistics V.27 (IBM Corp.) and R (version 4.5.1; R Foundation for Statistical Computing).

## Results

This study comprised 156 eyes (124 patients) that met the inclusion criteria and were included in the final dataset for analysis. Of these eyes, 28 (17.9%) were treatment-naïve; 14 (9%) were previously treated but had been off treatment for at least 6 months before Afl 8 injection. In the switcher group, 114 eyes (73.1%) had been pretreated with other anti-VEGF agents. We compared really treatment-naïve eyes with the eyes that had been stable off therapy for more than 6 months before developing new activity and found no relevant differences at the time of the first Afl 8 injection. Therefore, we pooled these 2 groups. Detailed patient demographics are presented in Table 1. Table 2 displays comparisons of baseline ocular characteristics between treatment-naïve and pretreated off-therapy eyes at the time of the first Afl 8 injection. The pretreatment characteristics of switchers are displayed in Table 3. Bilateral treatment with Afl 8 was applied to 32 patients (25.8%).

**Table 1:** Demographics and Baseline Characteristics of Naïve and Pretreated Eyes

Demographics	Naïve (n = 42, 26.9%)	Switchers (n = 114, 73.1%)
Age (yrs)	61.9 ± 10.7 (63 [55, 70])	64.6 ± 11.7 (67 [59, 71])
Sex (% female)	29%	34.4%
Diabetes type		
Type 1	2 (6.5%)	6 (11.8%)
Type 2	29 (93.5%)	45 (88.2%)
Insulin usage		
No	12 (38.7%)	31 (33.3%)
Yes	17 (54.8%)	60 (64.5%)
Missing	2 (6.5%)	2 (2.2%)
HbA1c	7.1 ± 0.9 (6.9 [6.5, 7.7])	7.0 ± 1.0 (7.0 [6.3, 7.3])

HbA1c = glycosylated hemoglobin.

Data are shown as mean ± standard deviation and (median [interquartile range]).

**Table 2:** Baseline Ocular Characteristics

Lens Status		
Pseudophakic	13 (31%)	67 (58.8%)
Phakic (until last visit)	28 (66.7%)	45 (39.5%)
Phakic (cataract surgery during observation) Unknown	1 (2.7%)	2 (1.8%)
VA (ETDRS letter scores)	72.9 ± 10.7	73.9 ± 11.2
	(75 [65.1, 80.2])	(75 [68.1, 80.2])
Central retinal thickness (μm)	413.7 ± 166.5	306.2 ± 103.7
	(409 [264, 509])	(286 [233, 345.5])
Central subfield thickness (μm)	448.9 ± 154.3	336.6 ± 90.5
	(436 [337, 527])	(317 [272.8, 366.5])
Macular fluid		
No fluid	4 (9.5%)	21 (18.4%)
IRF	30 (71.4%)	90 (78.9%)
Both IRF and SRF	6 (14.3%)	3 (2.6%)
Missing	2 (4.8%)	0
Diabetic retinopathy severity		
No apparent DR	8 (25.8%)	1 (1.1%)
Mild NPDR	0	20 (21.5%)
Moderate NPDR	7 (22.6%)	19 (20.4%)
Severe NPDR	12 (38.7%)	29 (31.2%)
PDR	4 (12.9%)	20 (21.5%)
High-risk PDR	0	4 (4.3%)
Prior laser treatment		
No	29 (69%)	40 (35.1%)
Focal macular	0	2 (1.8%)
Incomplete PRP	6 (14.3%)	30 (26.3%)
Complete PRP	7 (16.7%)	35 (30.7%)
Combined (1 + 2 or 1 + 3)	0	7 (6.1%)

DR = diabetic retinopathy; IRF = intraretinal fluid; NPDR = nonproliferative diabetic retinopathy; PDR = proliferative diabetic retinopathy; PRP = panretinal photocoagulation; SRF = subretinal fluid; VA = visual acuity.

**Table 3:** Pretreatment Features (Switchers Only)

Duration of previous treatments (yrs)	4.8 ± 3.6 (3.7 [2, 7])
Number of intravitreal injections prior to switch	31.1 ± 29 (20 [14, 41])
Last intravitreal treatment (n [%])	
Aflibercept	70 (61.4%)
Ranibizumab	13 (2.6%)
Faricimab	41 (36%)
Treatment interval prior to switch (wks)	7.6 ± 3.7 (6.5 [5, 9])
Reasons for switch (n [%])	
Persistent macular fluid on Q4W	47 (41.2%)
Fluid recurrence under extension	43 (37.7%)
Switch for potentially extended treatment intervals	20 (17.5%)
Other	4 (3.5%)

Q4W = every 4-wk interval.

A total of 101 eyes, including 14 treatment-naïve (Table S4, available at www.ophthalmologyscience.org) and 87 pretreated (Table S5, available at www.ophthalmologyscience.org), were excluded, with a follow-up of below 12 months and refusal to donate consent for the use of coded patient data as the main reasons.

**Figure 1:**
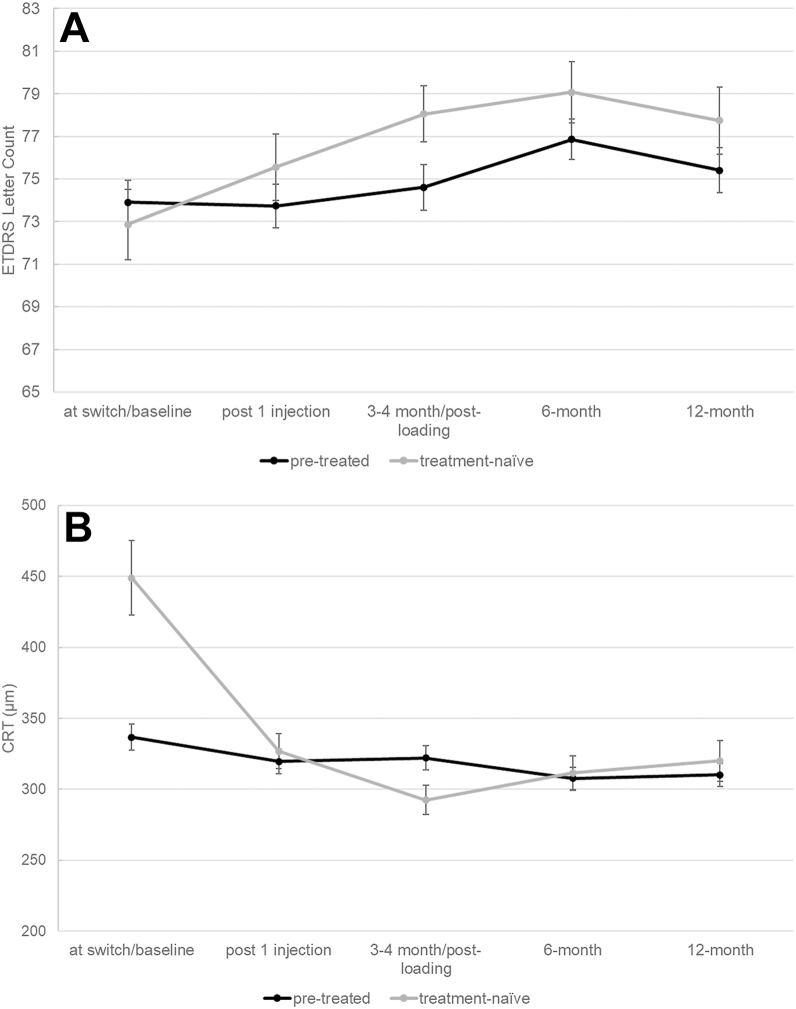
**A,** Changes in Snellen VA. **B**, Mean central subfield thickness (CST) in OCT scans of naïve and pretreated eyes from baseline to 12 months after switch to Afl 8. Central subfield thickness decreased in both treatment groups significantly at 12 months compared to baseline. While VA significantly improved in naïve eyes during the first year of Afl 8 treatment, it remained stable in pretreated eyes. Change in VA in pretreated eyes was not significant at any point in time. CRT = center-point retinal thickness; VA = visual acuity.

In both groups, VA improved over 12 months but did not reach significance in pretreated eyes (Fig 1A). Both treatment groups showed a significant decrease in CRT and CST from baseline to 12 months after beginning aflibercept (Table 6; Fig 1B).

**Table 6:** Development of CRT and CST over the First Year of Alf 8

Mean ± SD (median, 25%–75% IQR)	Treatment-Naïve		Pretreated	
	CRT	CST	CRT	CST
At baseline/switch	413.7 ± 166.5 (409, 364–509)	448.9 ± 154.3 (436, 337–527)	306.2 ± 103.7 (286, 233–346)	336.6 ± 90.5 (317, 273–367)
Post 1 injection	393.2 ± 75.2 (263, 243–361)	326.7 ± 70.0 (304, 279–384)	289.0 ± 98.4 (258, 225–338)	319.6 ± 85.8 (301, 269–347)
3–4 months	268.3 ± 76.8 (248, 216–348)	292.4 ± 58.5 (286, 252–349)	288.9 ± 92.8 (261, 231–324)	322.0 ± 80.7 (305, 269–349)
6 months	276.9 ± 86.2 (247, 220–350)	311.5 ± 70.0 (289, 263–368)	274.3 ± 84.6 (256.5, 221–307)	307.7 ± 73.0 (298, 255–333)
12 months	286.1 ± 104.2 (257, 210–365)	320.0 ± 80.1 (296, 267–379)	270.3 ± 80.3 (254, 210–308)	310.2 ± 69.9 (296, 257–343)

CRT = center-point retinal thickness; CST = central subfield thickness; IQR = interquartile range; SD = standard deviation.

### Treatment-Naïve Eyes

Treatment-naïve eyes experienced a mean VA improvement of +5.2 ± 10.4 letters (*P =* 0.006; n = 38) from a baseline VA of 72.9 ± 10.7 (median: 75, IQR 65.1–80.2) to 77.7 ± 9.7 (median: 80.2, IQR 72.2–85) letters 12 months after treatment initiation. Visual acuity at 12 months after initiating Afl 8 treatment was positively correlated with VA at baseline (*r*[38] = 0.51, *P =* 0.001). Change in VA from baseline to 12 months correlated with baseline CRT (*r*[38] = 0.44, *P =* 0.006) and CST (*r*[31] = 0.60, *P <* 0.001), as well as with any macular fluid (*r*[36] = 0.40, *P* = 0.017).

Visual acuity data from 1 eye undergoing cataract surgery during the observation period were censored from the time of cataract surgery to exclude bias due to cataract surgery. Two eyes (4.8%) gained ≥10 letters, and 10 eyes (23.8%) gained ≥15 letters.

**Figure 2:**
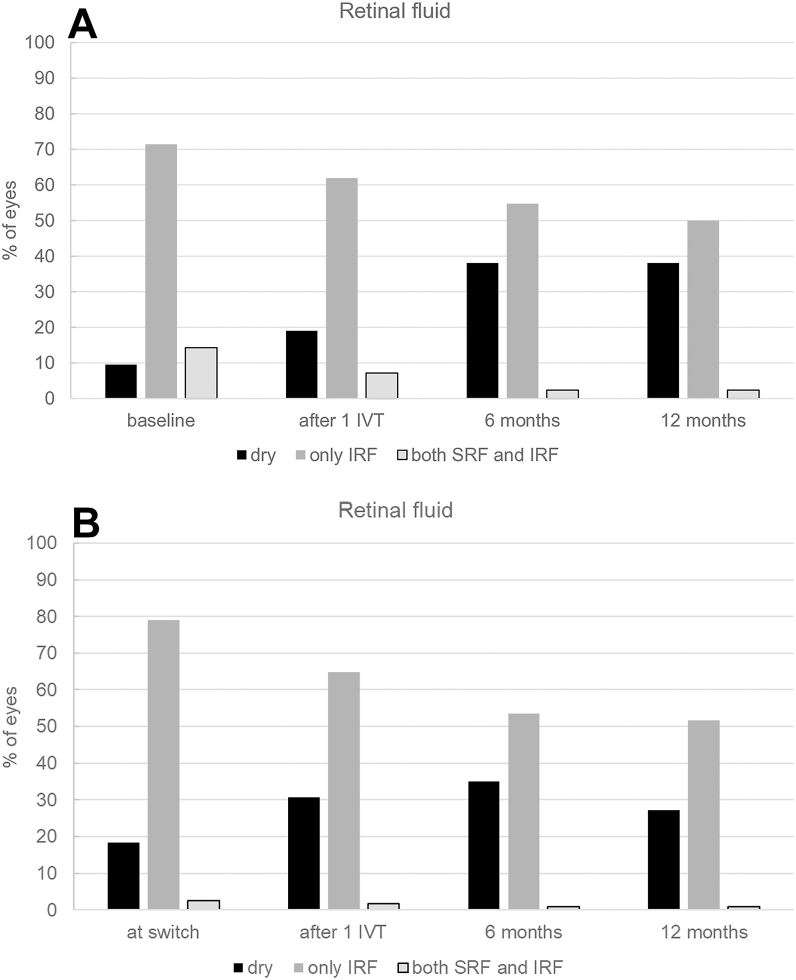
Macular fluid status after initiation of intravitreal Afl 8 in **(A)** treatment-naïve eyes and **(B)** eyes switched from any other anti-VEGF drug to Afl 8. Distribution of macular fluid and absence of fluid in OCT scans of naïve and pretreated eyes at baseline, after the first injection of Afl 8, after 6 months, and after 12 months. The proportion of eyes without macular fluid remarkably increased during the first year of Afl 8 treatment in both treatment groups. IRF = intraretinal fluid; IVT = intravitreal injection; SRF = subretinal fluid.

Macular fluid resolved in the central 1 mm of the ETDRS grid after a single Afl 8 injection in 4 eyes (9.5%), and after 12 months in 16 treatment-naïve eyes (38.1%, McNemar: *P* = 0.019; Fig 2A). Twenty-two eyes (52.4%) displayed persistent macular fluid 12 months after treatment initiation, of which 21 (50%) showed only IRF and 1 (2.4%) showed both IRF and SRF. When comparing treatment-naïve eyes with eyes that had been off therapy due to stability for at least 6 months between the last anti-VEGF injection and the first Afl 8 injection, 14 out of 28 treatment-naïve eyes (50%) showed persistent fluid, as did 8 out of 14 off-therapy eyes (57.1%; *P =* 0.50). The mean last treatment interval was 15.3 ± 12.0 (median: 12.0, IQR: 8–14) weeks, and the mean number of injections in the first year was 6.6 ± 2.3 (median: 7, IQR: 5–8).

Treatment with Afl 8 was interrupted in 2 eyes (4.8%) within the first year after complete resolution of retinal fluid, while 2 eyes (4.8%) were switched to faricimab and aflibercept 2 mg due to persistent macular fluid and patient preference, respectively.

### Pretreated Eyes

In pretreated eyes, VA moderately changed by +1.6 ± 7.9 letters (baseline 73.9 ± 11.2; median: 75.0 [68.1–80.2] ETDRS letters, 12 months after the switch 75.4 ± 10.1; median: 80.2, IQR: 69.9 – 83 letters (*P =* 0.094; n = 91; Fig 1A). As in treatment-naïve eyes, VA at 12 months after initiating Afl 8 treatment was positively correlated with VA at baseline (*r*[91] = 0.75, *P <* 0.001). The change in VA correlated with baseline CRT (*r*[91] = 0.30, *P =* 0.005) and CST (*r*[75] = 0.50, *P <* 0.001), but, in contrast to treatment-naïve eyes, not with the presence of macular fluid.

Visual acuity data from 2 eyes were censored from the time of cataract surgery to exclude bias due to cataract surgery. Three eyes (2.6%) gained ≥10 letters, and 6 eyes (5.3%) gained ≥15 letters.

Absence of fluid in the central 1 mm of the ETDRS grid was observed in 27.2%. A complete resolution of macular fluid was observed in 18 pretreated eyes (15.8%) after a single Afl 8 injection, and in 16 eyes after 12 months (14%, McNemar: *P* = 0.017). Of the 114 switched eyes, 60 (52.6%) showed persistent macular fluid at 12 months. Of these, 59 (51.8%) showed IRF, and 1 (0.9%) showed both IRF and SRF (Fig 2B).

**Figure 3:**
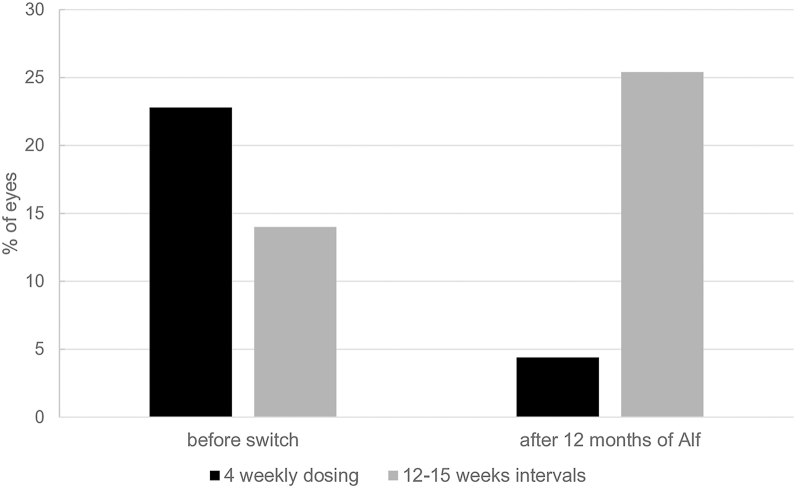
Distribution of treatment intervals of pretreated eyes at switch to Afl 8 and after the first year of Afl 8 treatment. The proportion of eyes with intervals of ≥12 weeks increased, while those with intervals of 4 weeks remarkably decreased.

The treatment interval changed from 7.6 ± 3.7 (median: 6.5, IQR: 5–9) weeks at switch to 13.0 ± 9.0 (median: 10, IQR: 6.7–15) weeks at 12 months (*P <* 0.001). The mean number of injections in the first year was 6.8 ± 2.6 (median: 7, IQR: 5–8), and the proportion requiring a 4-week dosing interval decreased from 22.9% to 4.4%. Meanwhile, the proportion achieving 12- to 15-week intervals increased from 14% to 25.4%. Figure 3 displays the distribution of treatment intervals 12 months after switch to Afl 8.

Information on the severity of diabetic retinopathy at the time of switching treatment was available for 31 treatment-naïve eyes and 93 pretreated eyes. Proliferative diabetic retinopathy (PDR) or high-risk PDR was reported in 4 treatment-naïve eyes and 24 pretreated eyes (Table 2). A subgroup analysis was performed to compare the treatment demand and fluid response after 12 months between eyes with PDR/high-risk PDR and milder forms of DR. The analysis showed no difference in the treatment demand under Afl 8. The number of injections received by the 24 eyes with PDR/high-risk PDR and the 69 eyes with milder forms of DR was similar (7.1 ± 2.5; median: 6.5, IQR: 5–9, compared to 6.7 ± 2.6, median: 7.0; IQR: 5–8; *P* = 0.54). The severity of DR also had no impact on treatment intervals 12 months after the switch (PDR/high-risk PDR: 12.4 ± 7.7, median: 10.0, IQR: 6.1–16.0, and in eyes with milder forms of DR, 13.2 ± 9.6, median: 11.0, IQR: 7–15; *P* = 0.93), nor on the presence of retinal fluid in the central 1 mm of the ETDRS grid (chi-square: *P* = 0.49).

Treatment was interrupted in 2 pretreated eyes (1.8%) due to stability (i.e., absence of retinal fluid). Meanwhile, 16 eyes (14%) were switched to another anti-VEGF agent due to persistent fluid: Treatment was interrupted in 2 pretreated eyes (1.8%) due to stability (i.e., absence of retinal fluid). Meanwhile, 16 eyes (14.0%) were switched to another anti-VEGF agent due to persistent fluid: 2 eyes were switched back to aflibercept 2 mg (1 after 2 episodes of mild IOI in response to Afl 8, one because of patient preference), 8 eyes (7.0%) were switched to faricimab and 6 eyes (5.3%) were switched to intravitreal dexamethasone.

### Safety Outcomes

One patient died for reasons not related to intravitreal treatment. No other systemic safety events were recorded. Six ocular safety findings were reported, all of which affected pretreated eyes (3.8%). Three of these cases experienced IOI (1.9%), one of these with 2 episodes (details see Table S6, available at www.ophthalmologyscience.org), 2 (1.3%) a rise in intraocular pressure, and 1 case of vitreous hemorrhage was documented (Table 7).

**Table 7:** Adverse Events

Adverse Events	Cases (n)
Intraocular inflammation	3
Rise in intraocular pressure	2
Vitreal hemorrhage	1

## Discussion

In this real-world cohort of DME patients treated with Afl 8 over a 12-month period, the findings of the Photon trial^2^ were reproduced, supporting efficacy and safety of Afl 8 in clinical routine. Meaningful VA gains were observed in treatment-naïve eyes, and significant reductions in CRT and CST were observed across all groups. A significant proportion of eyes could be extended to 12-16 weeks, which is a relevant advancement for their quality of life.^2^^,^^9^^,^^11^^,^^12^ The magnitude of VA improvement in pretreated or switcher eyes is more modest, underscoring the importance of initiating therapy timely for an optimal outcome.^13^^,^^14^ Persistent macular fluid is challenging, particularly in previously treated eyes and may require adjunctive or alternative therapies in refractory cases.^14^, ^15^, ^16^ The majority of the pretreated eyes in our study were difficult to treat, and they had a long treatment history with other anti-VEGF agents (Table 3). Our results suggest that systematic switching is worth trying, at least in patients with treatment intervals of 8 weeks or less, though not all patients respond equally well: 16 eyes (14%) required switching from Afl 8 to other agents. Notably, 7.9% of these eyes with high treatment demands reached longer treatment intervals and a 10-letter gain or more. Real-world studies typically report more modest visual gains than expected from clinical trials, particularly for previously treated eyes, which experience modest visual, but relevant morphological improvement.^17^, ^18^, ^19^, ^20^

Visual gains were positively correlated with baseline VA and were more modest in chronic/pretreated eyes. Treatment-naïve eyes gained an average of +4.9 letters, while pretreated eyes remained stable with an average of +1.6 letters at 12 months. A higher proportion of treatment-naïve eyes achieved a ≥15-letter gain. These results suggest a deterioration of retinal integrity over time in the presence of DME and support early switching to another agent in cases of incomplete fluid resolution. However, complete fluid resolution was achieved in only 31.1% of naïve and 21.1% of pretreated eyes at 12 months. It seems, thus, clear that, no matter how potent an anti-VEGF drug is or how often it is administered, only a minority of cases achieve complete drying during the first year if only VEGF inhibitors are used. Indeed, an ideal functional and morphological outcome must be balanced against the treatment burden, respecting patient needs and adherence to treatment. As expected, persistent fluid is common, especially in pretreated eyes. This was also seen with faricimab, in real-world settings with flexible retreatment criteria, where only 39% to 54% of DME eyes achieved a dry macula 12 months after switching. The correlation between baseline CRT/CST and VA change in our cohort reflects published experience.^2^^,^^21^^,^^22^

A key finding of the Photon trial^2^ is the potential for extended dosing intervals with high-dose aflibercept. In our study, the mean intervals were 15.3 weeks in naïve eyes and 13.0 weeks in pretreated eyes after 12 months. There was also a significant reduction in the proportion of patients requiring a 4-week dosing interval. This decreased the frequency of injections and clinic visits.^2^^,^^9^^,^^11^ Treat-and-extend regimens and real-world data further support the feasibility of extending the interval without compromising anatomic efficacy.^12^^,^^16^ As expected, compared to 2 mg standard dosing, increasing aflibercept dosage to 8 mg did not significantly improve functional outcomes for DME. This phenomenon was also reported in other real-world switching studies under other agents. However, it allowed to reach longer dosing intervals and reduced treatment burden.^18^^,^^23^

The safety profile of Afl 8 in this study is consistent with findings from the Pulsar (nAMD) and Photon (DME) trials. In the latter DME trial, 4 cases of IOI were observed beyond 491 eyes (0.8%) under Afl 8^2^. Despite the generally low incidence of AEs (3.8%) in our series, we observed 3 cases of IOI in pretreated patients (1.9%) under Afl 8, indicating an increased incidence in pretreated eyes, which occurred before the introduction of prefilled syringes. This finding is consistent with other recent reports.^5^^,^^6^^,^^9^ Although the safety data are thus generally reassuring, vigilance for IOI is warranted until further evidence has been gathered.^7^^,^^24^

In the Photon trial, the number of patients with IOI was low across all treatment groups (8q12 n = 4 [1%], 8q16 n = 0, and 2q8 n = 1 [1%]).^2^ With a safety profile reconfirmed in our cohort, which experienced six AEs in 156 eyes (3.8%; 124 patients) over more than 1000 injections, Afl 8 is an interesting addition to our anti-VEGF agent arsenal for treating DME, particularly in patients with high treatment demands. Nevertheless, recent real-world experience suggests an increased rate of IOI, which was manageable in all published and own cases and did not lead to permanent vision loss.^5^^,^^6^ Clearly, more long-term, real-world data are needed to confirm our findings and define the optimal patient population for high-dose therapy.

Despite its potential strengths, our study has limitations, such as its retrospective nature, the absence of standardized retreatment criteria, and possible inconsistencies in treatment decisions across different sites. One shortcoming is that we did not consider the duration and severity of the underlying diabetic disease, which may have impacted the functional and anatomical outcomes. Switzerland's health care context—namely, wide access to anti-VEGF drugs and intravitreal steroids; relatively good metabolic control in diabetic patients; flexible anti-VEGF labels; and broad cost coverage by compulsory basic insurances—may limit the generalizability of the findings to other populations. This study's real-world nature provides valuable complementary data to that from controlled clinical trials, which may be limited by their more homogeneous populations. Real-world settings include many types of patients (treatment-naïve patients, subjects with interrupted treatment, switchers with a stable situation or recalcitrant response), which complicates the interpretation of results. While the 12-month follow-up period offered valuable insights, it might not fully capture Afl 8's long-term efficacy and safety profile, especially considering the chronic nature of DME. High rates of residual edema, especially in pretreated eyes, may reflect the need for interval extension in the real world, but they have limited functional gains. Finally, the relatively small number of AEs indicates that this study is underpowered to detect new, relevant safety signals, though it is consistent with published safety profiles.

In conclusion, Afl 8 treatment results in significant anatomic and functional benefits for DME patients, especially those who have not received prior treatment. This enables extended treatment intervals for most patients without compromising safety. However, persistent fluid and modest visual gains in pretreated eyes demonstrate the difficulty of balancing visual potential and long-term morphological stability against patients' treatment burden in real life. Our results support the use of Afl 8 as a valuable treatment option. However, they also highlight the need for further research to improve the management of refractory cases with an individualized approach.
