# Supplementary material for: Twelve-Month Outcomes Using Aflibercept 8 mg in Treatment-Naïve and Pretreated Diabetic Macular Edema: A Swiss Retina Research Network Report
Source: Ophthalmol Sci. 2026 Jan 22;6(4):101087. doi: 10.1016/j.xops.2026.101087 (PMC12955161; doi:10.1016/j.xops.2026.101087)
Supplement: Table S4 [file mmc1.pdf]

Supplemental Table 4. Reasons for exclusion for each center in treatment-naïve patients

Center (number of included cases)	Follow up after first Afl 8mg of less than 12 months	Refusal to grant consent for the use of the patient's coded data	Dex injections less than 6 months before first Afl 8mg injection	Preexisting sructural damage to the macula from any other reason without functional potential	Snellen best-corrected visual acuity below 0.1 at diagnosis	Any intraocular surgery within 3 months prior to inclusion
Berner Augenklinik; n=12	1	2	-	-	-	-
Stadtspital Zürich; n=9	1	-	-	-	-	-
Augenärztepraxisgemeinschaft Gutblick; n=8	-	-	-	-	-	-
Kantonsspital St. Gallen; n=7	2	4	-	-	-	-
Istituto Neuroscienze cliniche della Svizzera Italiana; n=7	-	-	-	-	-	-
Vista Augenklinik Binningen; n=6	-	-	-	-	-	-
Hôpital ophtalmique Jules-Gonin; n=5	2	-	-	-	-	-
Swiss Visio Retina Research Center; n=3	-	-	-	-	-	-
University Hospital Zurich; n=2	2	-	-	-	-	-
Total	8	6	0	0	0	0
