# Supplementary material for: Twelve-Month Outcomes Using Aflibercept 8 mg in Treatment-Naïve and Pretreated Diabetic Macular Edema: A Swiss Retina Research Network Report
Source: Ophthalmol Sci. 2026 Jan 22;6(4):101087. doi: 10.1016/j.xops.2026.101087 (PMC12955161; doi:10.1016/j.xops.2026.101087)
Supplement: Table S5 [file mmc2.pdf]

Supplemental Table 5. Reasons for exclusion for each center in patients switching from another anti-VEGF to Afl 8mg

Center (number of included cases)	Follow up after first Afl 8mg of less than 12 months	Refusal to grant consent for the use of the patient's coded data	Dex injections less than 6 months before first Afl 8mg injection	Preexisting structural damage to the macula from any other reason without functional potential	Snellen best-corrected visual acuity below 0.1 at diagnosis	Any intraocular surgery within 3 months prior to inclusion
Berner Augenklinik; n=38	3	16	1	1	1	-
Kantonsspital St. Gallen n=16	2	3	1	-	-	-
Augenärztepraxis-gemeinschaft Gutblick; n=14	2	6	-	1	1	-
Vista Augenklinik Binningen; n=9	3	2	-	-	-	-
Stadtspital Zürich; n=9	11	5	1	-	1	-
University Hospital Zurich; n=8	8	5	-	1	-	1
Istituto Neuroscienze cliniche della Svizzera Italiana; n=5	-	-	1	-	-	-
Pallas Kliniken; n=5	-	1	-	-	-	-
Swiss Visio Retina Research Center; n=4	-	-	-	-	-	-
Hôpital ophtalmique Jules-Gonin; n=3	7	-	-	-	-	1
Universitätsspital Basel; n=2	-	-	-	-	-	-
Talacker Augenzentrum TAZZ; n=1	1	-	-	-	-	-
Total n (exclusions)	37	38	4	3	3	2
