# Supplementary material for: Twelve-Month Outcomes Using Aflibercept 8 mg in Treatment-Naïve and Pretreated Diabetic Macular Edema: A Swiss Retina Research Network Report
Source: Ophthalmol Sci. 2026 Jan 22;6(4):101087. doi: 10.1016/j.xops.2026.101087 (PMC12955161; doi:10.1016/j.xops.2026.101087)
Supplement: Table S6 [file mmc3.pdf]

Supplemental Table 6. Detailed information about the intraocular inflammation (IOI) episodes

	**Patient 1**	**Patient 2**	**Patient 3 (first episode, after first injection)**	**Patient 3 (second epidsode, after 5th injection)**
Location and severity of Inflammatory changes	Anterior chamber cells (+)-+	Anterior chamber and anterior vitreous cells ++	Anterior chamber cells +	Anterior cells +, fine endothelial precipitates
Vasculitis	No	No	No	No
Management	Dexamethasone 0.1% eye drops 5x/day for 3 days, 4x/day for 5 days, then tapering over 15 days (3x/2x/1x for 5 days each)	Peroral Prednisone, beginning with 1mg per kg of body weight per day, halfening of dose every 2 days. Topically, prednisolone acetate 1% 8x/day, thereafter tapering every 3-5 days over 4 weeks; Hydrocortisone 1% ointment 1x/day for 2 weeks; scopolamine 3x/day for 5 days.	Prednisolone acetate 1% 4x/ day for 3-4 days, tapering over 2 weeks	Prednisolone acetate 1% 4x/ day for 3-4 days, tapering over 16 days
Outcomes	VA drop from 0.5 to 0.1 (at diagnosis of IOI), full recovery after resolution of inflammation	VA drop from 0.5 to 0.12 (at diagnosis of IOI), full recovery after resolution of inflammation	No change in visual function due to IOI	Visual acuity stable until resolution of IOI
Impact on treatment decisions	no	Intravitreal injections stopped	No	no
Treatment switch	No	Treatment stopped beyond data lock	No	Switch to Afl 2mg
